# An Acute Jejunojejunal Intussusception Revealing a Metastatic Combined Lung Cancer

**DOI:** 10.1155/2021/9999605

**Published:** 2021-05-25

**Authors:** Feryel Letaief-Ksontini, Ryma Boujnah, Yosra Yahiaoui, Yosra Zaimi, Meriem Ksentini, Raoudha Aloui, Khedija Meddeb, Amel Mezlini

**Affiliations:** ^1^Medical Oncology Department, Faculty of Medicine of Tunis, Salah Azaiez Institute, El Manar University, Tunis, Tunisia; ^2^Gastroenterology Department, Faculty of Medecine of Tunis, Cherles Nicolles Hospital, El Manar University, Tunis, Tunisia; ^3^Histopathology Department, Faculty of Medicine of Tunis, Charles Nicolles Hospital, El Manar University, Tunis, Tunisia

## Abstract

Intussusception is a relatively common disease in pediatric age but it is uncommon in adults. We report a case of a 49-year-old male who presented with an acute jejunojejunal intussusception revealed by abdominal pain and vomiting. He underwent an en bloc resection, and pathological findings concluded to a metastasis of a pulmonary combined small cell carcinoma and adenocarcinoma. A subsequent CT scan revealed the primitive mass of the right lung with no evidence of secondary localization. The biopsy was difficult to perform. The patient underwent a pneumonectomy with lymph node dissection confirming the same diagnosis. He made a good recovery from the surgery, and a postoperative chemotherapy was administrated, and he is in remission until this date.

## 1. Introduction

Intussusception is a relatively common disease in pediatric age but it is uncommon in adults. It represents only 1% to 3% of causes of bowel obstruction in an elderly population, and it most often requires surgery [1, 2]. In the majority of cases, intussusception is caused by a bowel disease, and about 50% of these lesions are malignant. However, intussusception due to a jejunal metastasis of combined lung cancer is extremely rare [3]. Indeed, lung cancer metastasis to the bowel is uncommon, reported in less than 1% of patients [4]. In this present case, we report the observation of a patient who presented with an acute intestinal intussusception caused by a jejunal metastasis of pulmonary combined small cell carcinoma and adenocarcinoma and we present a review of the literature.

## 2. Case Presentation

We report the case of a 49-year-old man, with unremarkable past medical and family history but with an 80 pack-years of smoking. He was admitted in the Surgery Department in June 2020 for an acute intestinal obstruction revealed by an abdominal pain with vomiting. An abdominal computed tomography scan (CT scan) was performed in emergency and concluded to an intussusception upstream of a well-limited tumor with no sign of intestinal distress.

**Figure 1:**
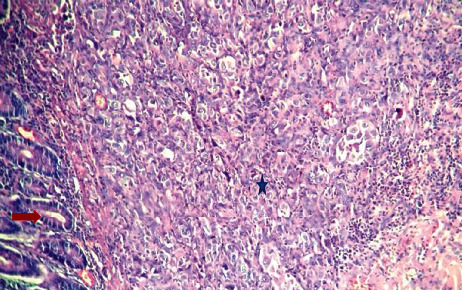
Large undifferentiated carcinomatous cells (blue star) infiltrating the small intestine wall; normal mucosal glands at the left bottom (red arrow) HE ×100.

The patient was operated in emergency. The intraoperative findings showed a jejunojejunal intussusception located 1 meter from the duodenojejunal angle related to a well-limited 3 cm tumor. A resection of 10 cm of the intestine including the mass was performed with an end-to-end anastomosis. Pathologic examination revealed a pT2 undifferentiated carcinoma with a sarcomatoid component involving all layers of the jejunum and expressing only cytokeratin 7. Cytokeratin 20, CD34, thyroid transcription factor-1, CD117 (c-kit,) and HMB45 were all negative (Figure 1).

**Figure 2:**
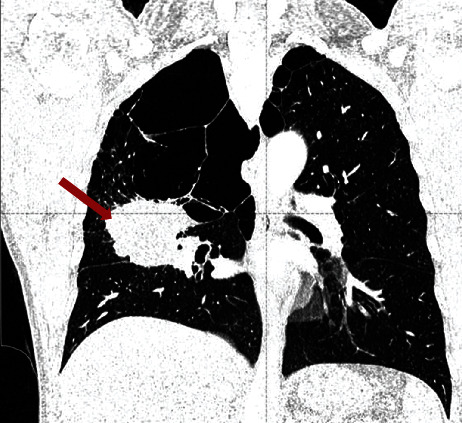
CT scan image of the right lung tumor (red arrow) extending to the three lobes with interbronchial lymph nodes.

A subsequent thoracic CT scan was performed to complete the extension report, which showed a polylobed mass extending to the three lobes of the right lung measuring 6 cm, with right interbronchial lymph nodes (Figure 2). No further evident metastases were revealed. A fibroscopy showed an inflammatory intersegmental spur of the right lobe; biopsy was negative. Transparietal biopsy was difficult to perform. The patient was operated; intraoperatively, there was a large extension of the mass of the right lung in the fissure making arterial dissection at this level impossible so a lobectomy could not be performed. The patient had a pneumonectomy with lymph node dissection. Postoperative recovery was eventful in the patient.

**Figure 3:**
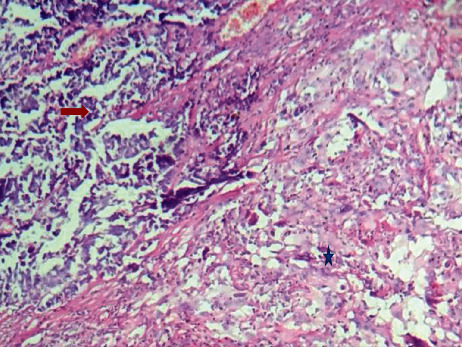
Pulmonary carcinoma: at the left top: small cell component (red arrow); at the right bottom: solid large cell component (blue star), HE ×100.

Histological examination revealed a combined lung tumor appearance with a predominant component (80%) consisting of a small cell neuroendocrine carcinoma associated with a solid adenocarcinomatous component, with massive infiltration of the lung, rupture of the visceral pleura, and extension to parietal fat tissue but with free surgical margin at this level and without sarcomatoid component. Vascular invasion and spread through air spaces were also noted (Figure 3).

Lymph dissection revealed one involved lymph node among the 23 examined. Immunohistochemistry showed a positivity of the small cell component for CD56 and chromogranin.

After review of the 2 tumors, the pathologist concluded to a jejunal metastasis of a pulmonary combined small cell carcinoma and adenocarcinoma.

The patient was diagnosed with combined lung cancer stage IV, and the decision was to perform postoperative chemotherapy with cisplatin 30 mg/m^2^ per day (D) at days 1, 2, and 3 associated with etoposide 80 mg/m^2^ per day at D1, D2, and D3 and Navelbine 30 mg/m^2^ per D at D1 every 21 days + granulocyte colony-stimulating factor (G-CSF) [D1 = D21]. The patient achieved 4 courses of chemotherapy with good tolerability and is currently in remission.

## 3. Discussion

This case highlights the potential for the lung cancer to metastasize to the gastrointestinal tract (GI). These metastases are very rare with a poor prognosis. Frequency is only about 0.2–1.7% [4, 5]. A higher prevalence of 4.7–14% was described in other autopsy studies. The jejunum represents the most common site followed by the ileum then the duodenum [6, 7].

In our case, the metastasis was revealed by an intussusception which is rare since the majority of causes of small bowel intussusceptions are from benign lesions. Malignancy is found in 6–30% of cases following surgical resection. It includes primary lesions such as GI tumors or lymphoma and also metastases, particularly from melanoma, breast cancer, and lung cancer [1, 8, 9]. Non-small-cell lung cancer or small cell cancer metastases to the bowel are rarely reported in the literature. To the best of our knowledge, no such case has been reported to date with a combined lung cancer.

The pathophysiological mechanism of the occurrence of these metastases has not yet been well established. Some hypotheses suggest a hematogenous spread and others a lymphatic spread through the mediastinum and the retroperitoneum to the mesentery [10, 11].

Symptoms of gastrointestinal tract metastases are often nonspecific, which explains their difficult and late diagnosis. In the majority of cases, the most common symptoms are nausea, abdominal pain, vomiting, constipation, gastrointestinal bleeding, or weight loss [12, 13]. Intussusception is a rare clinical presentation.

Treatment of these lesions is still challenging, and there is no consensus on the management of GI metastases of lung cancer. Researchers described that the presence of a mass in CT scan with associated presence of neoplasms certainly warrants surgical treatment [8]. Surgical intervention is necessary in almost every case because of frequent complications including hemorrhage, perforation, or, obstruction [3].

The prognosis of GI metastasis is very poor, about 2 to 4 months [4, 14]. However, there are reports that show better survival of more than 2 years following small bowel resection, but this situation remains rare [15]. Finally, there is also no consensus in the postoperative treatment of these tumors. In our case, the patient was considered oligometastatic and a postoperative chemotherapy was administrated.

In conclusion, the majority of cases of intussusception should be treated with surgery. Timing of the surgery depends on the severity of the clinical symptoms. Furthermore, clinicians should also discuss a metastatic spread in the presence of a background of malignancy and carefully explore the intra-abdominal organs in order to avoid overlooking the possibility of multiple metastases.
